# The surgical treatment strategies for thoracolumbar spine fractures with ankylosing spondylitis: a case report

**DOI:** 10.1186/s12893-019-0565-x

**Published:** 2019-07-26

**Authors:** Yang Min, Gu Hui-Yun, Zhong Hou-cheng, Xie Yuan-long, Jin Wei, Cai Lin, Wei Ren-xiong

**Affiliations:** Department of spine, Zhongnan Hospital of Wuhan University, Donghu Road NO.169, Wuhan, Hubei 430071 People’s Republic of China

**Keywords:** Ankylosing spondylitis, Spine fractures, Osteoporosis, Surgery, Postoperative complications, Postoperative management

## Abstract

**Background:**

Ankylosing spondylitis (AS) is a chronic inflammatory disease that primarily affects spine and paraspinal soft tissue. Ankylosing spondylitis is one of the causes of osteoporosis and patients with ankylosing spondylitis tend to have spinal fractures due to limited mobility and osteoporosis. In recent years, due to the increase in the number of patients with AS, patients with AS and thoracolumbar spine fractures have gradually increased. In the past 1 year, we have treated 3 cases of AS with thoracolumbar spine fractures via simple posterior internal fixation and this paper aims to report its clinic effect.

**Case presentation:**

All the three patients selected had a history of ankylosing spondylitis for nearly 30 years, and one of them developed a thoracolumbar spine fracture after falling when he walked, and the other two developed a thoracolumbar spine fracture without any reason. They were hospitalized for “low back pain” and were diagnosed as fractures after careful physical examination and imaging examinations such as X-ray, CT, and MRI. After the preoperative preparation was completed, all the three patients underwent surgery with simple posterior internal fixation-reduction of the fracture and pedicle screw fixation via posterior approach. All the implants-pedicle screws and connecting rods-are made of titanium alloy. For postoperative management, we asked all the patients to stay in bed for 3 weeks after the operation, and then slowly move down with the help of crutches. Fracture healing and neurological function recovery were observed postoperatively. All the three patients recovered satisfactorily after surgery, and the follow-up confirmed that the fracture healed successfully after 3 months.

**Conclusions:**

The 3 patients included 2 men and 1 women. All the 3 patients recovered well after surgery, and the follow-up confirmed that the fracture healed successfully after 3 months. One man developed urination dysfunction after operation and recovered to normal 3 months after rehabilitation exercise.

## Background

Ankylosing spondylitis (AS) is a chronic inflammatory disease that primarily affects spine and paraspinal soft tissue. Chronic inflammation and new bone formation lead to pathological remodeling of the spine [1]. In the late stage of ankylosing spondylitis, ligament calcification at the edge of the spine and bone fusion between the vertebral bodies make a “bamboo-like spine”. This “bamboo-like spine” is less active and less elastic; and osteoporosis is usually associated with ankylosing spondylitis, making the spinal prone to fracture. Kyphosis, another characteristic of AS, usually results in a limited field of vision, a reduced balance, and an increased risk of falls and fractures [2, 3]. Usually, a minor trauma, even no trauma, can lead to a spinal fracture [4]. The bamboo-like spine tends to form the characteristics of long bones, which makes the fractures of the spine unstable, similar to the characteristics of long bone fractures. Thus, the risk of spinal cord injury increased after spinal fracture with AS. Therefore, operation should be performed in time to reduce the risk of spinal cord injury [5, 6]. However, because of calcification and vertebral rotation, the anatomy of the vertebral body in patients with AS is not the same as normal, which leads to the difficulty of pedicle screws placement. The risk of operation and postoperative complications in patients with ankylosing spondylitis is much higher than that in normal people [7–9]. Ankylosing spondylitis with thoracolumbar spine fractures is not very rare, but we have treated 3 such patients, two of whom were completely unhurt, and suddenly suffered from low back pain without a reason. After the CT and MRI examination, it was found to be a fracture. Here we want to report the development of these three interesting cases and the effect of our treatment.

## Case presentation

We retrospectively analyzed 3 patients (two men and one woman) with AS and thoracolumbar spinal fractures who underwent surgery in our hospital between 2016.03 and 2017.03. All the three patients were hospitalized for “low back pain” and were diagnosed as fractures after careful physical examination and imaging examinations such as X-ray, CT, and MRI. PE: There is tenderness in the lower back of all the three patients. All the patients fulfilled the modified New York criteria [10, 11] for primary AS and all the three patients had a history of ankylosing spondylitis for about 30 years. After hospital admission, CT, MRI, X-ray were taken and all HLA-B27 were detected to be positive. And the fractures of these three people are all type IIIB according to the Dennis classification. One male had a history of falling down before admission, and the other two complained of no history of falling down or any other trauma. According to the American Spinal Injury Association (ASIA) grading system, the spinal nerve function of the man who had a history of falling down was classified as class D, that of the other two were classified as class E (Table 1).

**Table 1:** Clinical data of the patients in this study

Case No.	Age(y)	Sex	Level of fracture	Mechanism of injury	Treatment	ASIA^a^ grade preoperatively	ASIA grade postoperatively
1	63	M	T12-L1	Falling	Pedicle screw fixation (T10-L3)	D	E
2	51	W	L1-L2	No trauma	Pedicle screw fixation (T11-L3)	E	E
3	56	M	T10-T11	No trauma	Pedicle screw fixation (T9-T12)	E	E

^a^*ASIA* American Spinal Injury Association

**Fig. 1:**
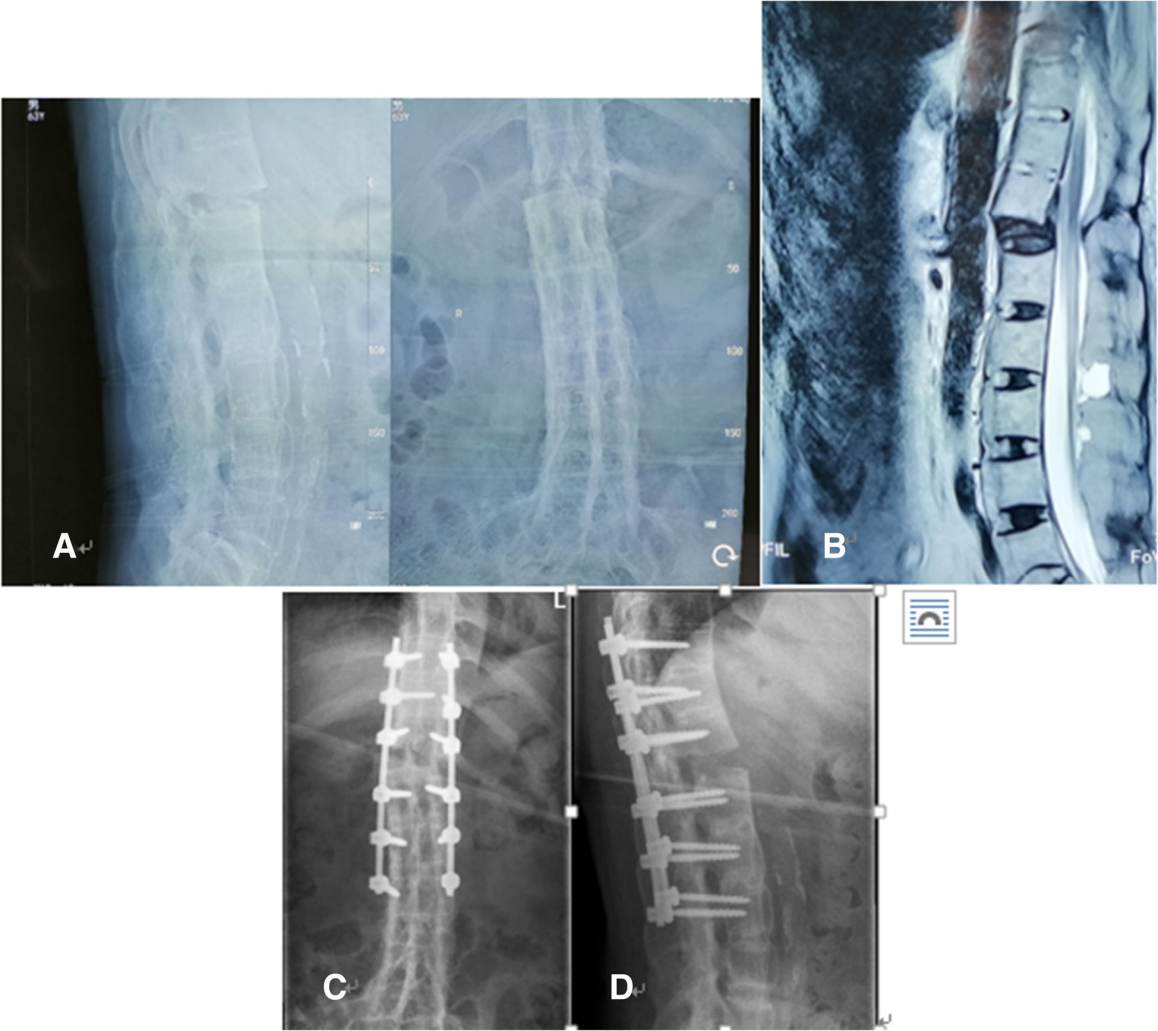
The patient had a 40-year history of AS and was admitted to the hospital due to a fall. **a** Preoperative X-ray shows an obvious intervertebral bone bridge made a “bamboo-like spine”, and the fracture end is obviously displaced. **b** Preoperative MRI also shows the fracture end is obviously displaced. **c**-**d** Postoperative X-ray shows the second pedicle screw on the right side did not meet the ideal position, resulting in the urinary dysfunction after operation, which recovered 3 months after discharged. And his neurological function was improved from degree D to degree E 1 year after surgery

**Fig. 2:**
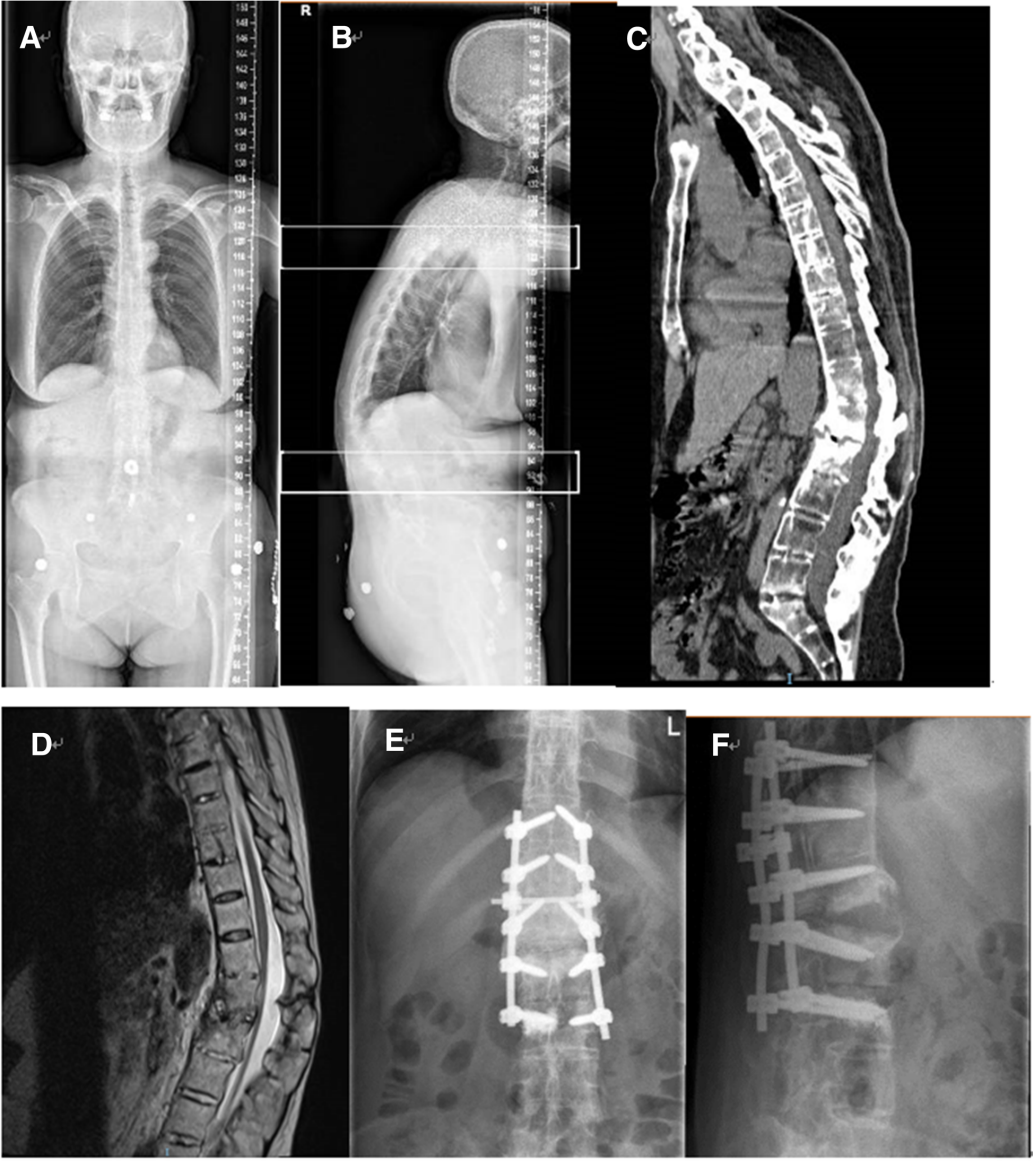
This patient had a history of AS for more than 36 years, with severe kyphosis. He was admitted to the hospital due to low back pain, without any obvious trauma. There was no spinal cord injury before surgery. **a**-**b** Preoperative X-ray shows a severe kyphosis. **c** Preoperative CT shows obvious vertebral calcification and osteoporosis, the fracture position was L1/2. **d** Preoperative MRI shows significant signal changes between L1/2. **e**-**f** Postoperative X-ray shows satisfactory pedicle screw position and good resetting effect

**Fig. 3:**
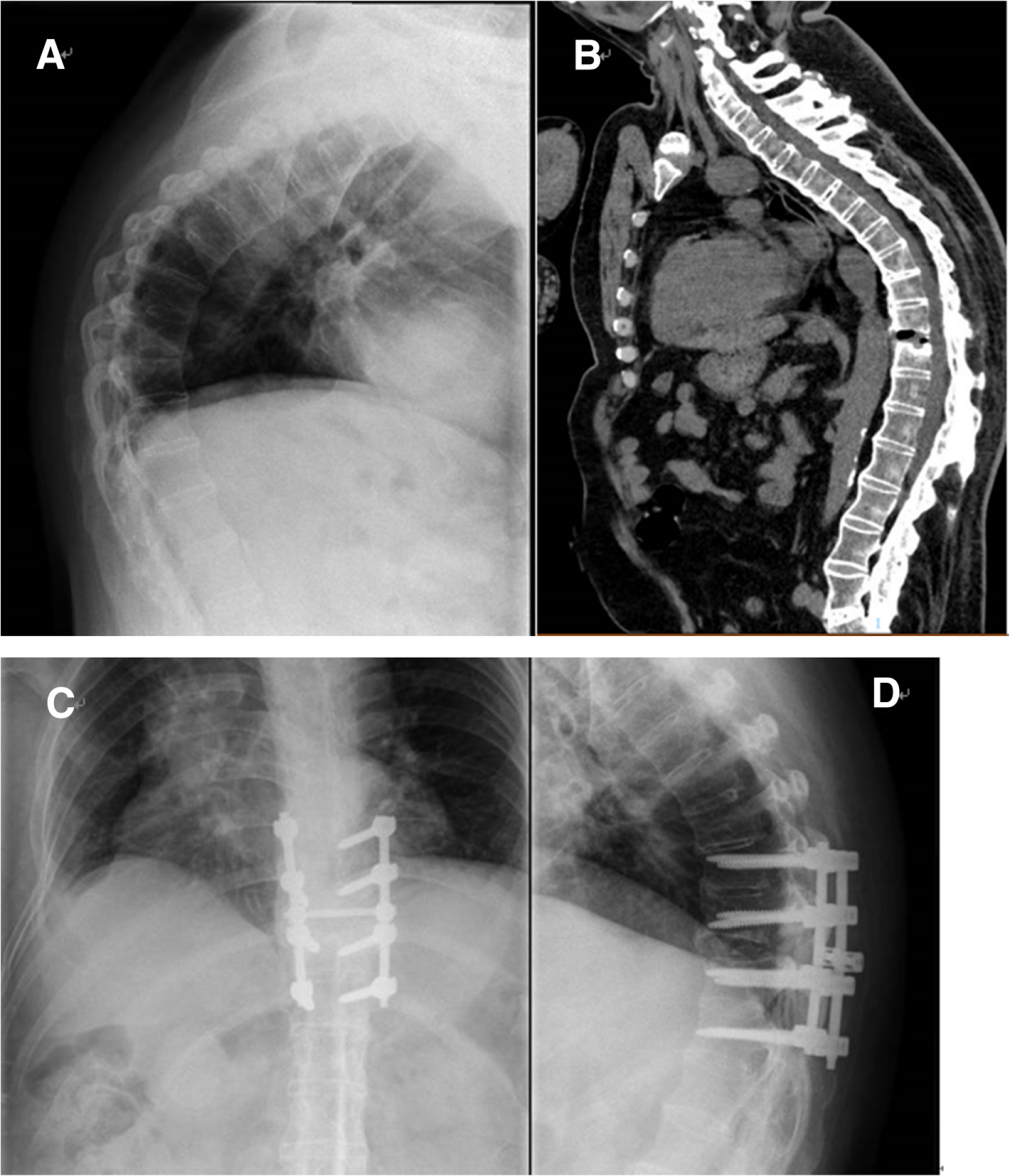
This patient had a history of AS for about 38 years, with severe kyphosis. He was admitted to the hospital due to back pain, without any obvious trauma. There was no spinal cord injury before surgery. **a** Preoperative X-ray shows an obvious severe kyphosis. **b** Preoperative CT shows obvious vertebral calcification and the fracture position is T10/11. **c**-**d** Postoperative X-ray shows satisfactory pedicle screw position and good resetting effect

Of the three patients, the first patient was a 63-year-old man who had a fracture after a fall and the fracture was between the T12-L1 segments. On MRI and X-ray, there were obvious fractures between T12 and L1, accompanied by displacement (Fig. 1). The patient did not have any symptoms of neurological deficits before the fall, but the muscle strength of the right lower limb decreased after the fall. After comparing the images of MRI, we believe that the right lower limb muscle strength decreased due to the spinal nerve injury caused by the dislocation after the fracture. After X-ray, the position of the pedicle screw on the right side of T11 was found to be not ideal, and the patient developed urinary dysfunction after surgery. After our discussion, we found that the risk of the second operation was too high, so we only gave him 2 weeks of neurotrophic therapy and bladder function rehabilitation exercise (rehabilitation exercise is done by a dedicated rehabilitation center). Our follow-up found that the patient’s urinary function returned to normal at the third month after surgery, and the muscle weakness of his right lower extremity recovered 1 year later. The second patient, a 51-year-old woman, suffered from a fracture without any trauma. The fracture segment was between L1 and L2. On CT, obvious calcification and osteoporosis can be seen and fractures occur in calcified soft tissue between L1 and L2 (Fig. 2). We performed posterior pedicle screw fixation for T11-L3. She recovered well after the operation, and the fracture healed at the 3rd month after the operation. The third patient was a 56-year-old man who had a fracture without any trauma. The fracture segment was between T10 and T11 (Fig. 3). Preoperative CT showed slight displacement between T10 and T11 and the main site of fracture was calcified ligament between T10 and T11. We performed T9-T12 posterior pedicle screw fixation on him, and he recovered very well.

### Surgical method

All the three patients underwent simple posterior pedicle screw fixation surgery after correcting hypoproteinemia. The patient was placed in a prone position after general anesthesia was administered and then conventional disinfection and draping were performed. A C-arm was used to position and mark the diseased vertebra, and then make an approximately 10 cm long surgical incision centered on the diseased vertebra. The skin, subcutaneous tissue, and muscular fascia were stripped layer-by-layer until we can see the articular processes and vertebral lamina. Thereafter, insert the pedicle screws in the diseased vertebra and at least two upper and lower segments, then install the orthopedic round stick, and slowly reset the diseased vertebra. It can be seen that all three patients had obvious soft tissue calcification and vertebral bone hyperplasia, and the intervertebral space was very narrow. These factors made the placement of pedicle screw very difficult. For these reasons, the position of a pedicle screw in one patient was not ideal. A C-arm was used to determine the position of pedicle screws and the effect of resetting. Then, fully flush the surgical incision, place a drainage tube on each side of the incision, and suture the incision layer by layer. All the 3 operations were successfully completed.

### Post-operative management

We asked all the patients to stay in bed for 3 weeks after the operation, and then slowly move down and walk with the help of crutches. The man who developed urinary dysfunction was given 2 weeks of neurotrophic therapy and bladder function rehabilitation exercise. All the three patients were given routine post-operative care and treatment, including fluid and electrolyte regulation, blood pressure stabilization, and correction of hypoproteinemia and anemia. VAS scores were recorded before and after operation.

### Outcome

One patient developed urinary dysfunction after operation while the other two had no postoperative complications. On the third day after surgery, X-ray showed that the patient with urinary dysfunction had a pedicle screw that did not meet the ideal position. The remaining two patients had satisfactory pedicle screw position and good resetting effect (Figs. 1, 2, 3). All the patients were asked to return to our hospital to review an X-ray in the 1st、3rd、6th month after discharged to determine whether the pedicle screws were loose and check the healing of the fracture. All the three patients were asked to come to the hospital for a review every month in the first 3 months, and then every 3 months thereafter. And every month we call to inquire about the patient’s current status.

After surgical treatment, the fractures healed in all patients, and other surgery-related complications were not observed in the follow-up period. The patient who developed urinary dysfunction after surgery recovered in the third month after discharged, and his neurological function was improved from degree D to degree E 1 year after surgery. For the first patient, the preoperative VAS score was 6. At the first month after operation, the VAS score was reduced to 2, and at the third month after operation, it was reduced to 0. The second patient had a preoperative VAS score of 5.5, which was reduced to 2 at the first month of follow-up and 0 at the third month of follow-up. For the third patient, the preoperative VAS score was 5. The VAS score dropped to 1 point 1 month after the operation and to 0 point 3 months after the operation.

## Discussion and conclusions

With the progression of AS, the two major pathological features (chronic inflammation and new bone formation) continue to occur in various segments of the spine, and the calcification of the vertebral and paraspinal ligaments will become more and more serious. In the later stage of the disease, the “bamboo-like spine” is going to form a long bone-like feature and this feature will make the spinal fracture unstable and easily affect all the three columns of spine [12], which is easy to cause spinal cord injury [13]. Patients with AS are often accompanied by osteoporosis in the later stages of the disease [14], and spinal fractures can occur without violence or with slight violence. Patients with AS are often accompanied by kyphosis, which make the patients’ center of gravity tilted forward, thus the patients’ balance ability will decrease, as a result, it will increase the risk of falls, and reduce the patients’ ability to self-protect during falls [1]. All these factors can increase the fracture risks in patients with AS. Some studies have reported [15], the incidence of osteoporosis in patients with AS for the past 10 years is 25%, and the incidence of spinal fracture is about 10%. The stress on the thoracolumbar spine is usually larger, and the incidence of fractures in the thoracolumbar segments is higher [16, 17]. Our study, through long-term follow-up of 3 patients with AS and thoracolumbar spine fractures, found that simple posterior internal pedicle screw fixation surgery has a good effect on such fractures. During the follow-up period, no obvious postoperative complications, such as pedicle screws retraction, broken nail, loosening, nonunion of fracture, were observed. All the three patients successfully recovered to the state of life before the injury.

Fractures are always difficult to be found in the early state in the patients with AS [18], especially those with no obvious trauma. Because the pain in the early stage of the fracture is difficult to distinguish from the inflammatory pain of AS, and patients always tend to choose to stay at home rather than go to hospital for an examination, especially when they have no obvious trauma. When going to a hospital, patients always take an X-ray first, but X-ray is a 2D picture and it may sometimes miss some fractures that have no obvious displacement. After ossification of spinal ligaments, the ossified ligaments can also be “fractured” [19], and this “fracture” is also one of the important causes of spinal instability and continuous back pain. X-ray is difficult for early fracture diagnosis while CT and MRI play a very important role in the diagnosis of fractures [20], and MRI can show the signal changes of the spinal cord and the volume of the spinal canal. The contents that MRI delivers will be an important indication to determine whether the laminar decompression is needed.

Surgical treatment should be performed as soon as the spinal fractures with AS is diagnosed [21]. Although surgical treatment also has certain risks of surgery related complications, the risk of non-surgical treatment will be greater [22], so unless the risk of surgery is unacceptably high, it is generally recommended to perform the surgery treatment as soon as possible. For patients with AS have poorer bone condition, the time that conservative treatment needs will be longer than normal people of the same age need, and the risk of the complication of bed rest is greater, so conservative treatment is not recommended. Some scholars have reported [23] that surgical treatment can significantly improve the survival rate of patients with spinal fractures in AS. Westerveld et al. [24] pointed out in their meta-analysis that timely surgical treatment can improve neurological function and reduce the incidence of overall complications.

Ankylosing spondylitis with spinal fractures can be performed with simple anterior approach, simple posterior approach, and anterior-posterior approach. Both simple anterior and simple posterior approaches are single-cortical fixations, similar to long bone fractures. Because of thoracic and abdominal organs and large vessels in front of the spine, anterior surgery is more difficult [25], and the holding force of the screw in the anterior approach is always insufficient, so the anterior approach is mostly used to restore the front column [26], especially when the front column of spine was severely collapsed and difficult to be restored by posterior approach. Anterior and posterior combined surgery has the advantages of the best reduction and the most powerful holding force, but it has a large trauma, a long operation time, and a high risk of complication for elderly patients with poor basic conditions, so the anterior and posterior combined surgery approach is not wildly used actually [27]. Young patients with good basic conditions and severe fractures in all the three columns can try the combined anterior and posterior surgery approach. Simple posterior surgery is the most widely used method currently [24]. Most people choose the simple posterior approach because this approach, by inserting pedicle screws, has a powerful holding force, a good effect on reduction and good postoperative stability. Most importantly, it has less trauma. Because kyphosis deformity is common in the late stage of AS, the posterior column is the tension side, and the anterior column is the pressure side. With reference to the experience of internal fixation of the extremity fracture, the internal fixation is more stable when placed on the tension side [25]. This is also one of the reasons why the simple posterior approach is accepted by more people. Bredin et al. [28] reported a percutaneous surgery that also achieved good clinic effect for patients with ankylosing spondylitis and spinal fractures, and this method further reduced surgical trauma. All the three patients included in our study were treated with pedicle screws internal fixation by simple posterior approach and the follow-up results showed this approach had a good clinical effect on spinal fractures with AS. Kurucan E et al. also found that the best surgical method for thoracolumbar fracture patients with AS is posterior internal fixation [4]. This is consistent with our conclusions from these three cases.

Due to the long-term chronic inflammation and the calcification of the surrounding ligaments, the elasticity of the vertebral may decrease, and even the anatomical structure may be somewhat different. Compared with the normal vertebral, it is more difficult to insert the pedicle screws in the vertebral of AS. When placing the pedicle screws, more attention should be paid to identify the anatomical structure and it’s best to successfully insert the pedicle screws the first time we try. In patients with AS and spinal fractures, the number of vertebral segments in which pedicle screws are placed is currently controversial. Kruger et al. [29] reported that only 1.8 segments above and below the injured vertebra need to be fixed and reconstructed on average, but Yeoh et al. [30] confirmed that three segments at least need to be fixed above and below the injured vertebra. Of all the 3 patients included in this study, 2 segments above and below the injured vertebra were fixed and it turned out it has a good clinic effect.

In summary, through these three patients, we believe that AS patients with thoracolumbar spine fractures should be operated as early as possible. We performed posterior pedicle screw fixation in all three patients, and fixed the two upper and lower segments of the fractured vertebral body. The follow-up proved that the effect was very good.

There are also some shortcomings in this report. The number of the reported cases is too small, and no statistical comparison have been conducted. The next study will further expand the number of cases and further compare the effects of all the surgical methods for spinal fractures with AS.

## Data Availability

The data and materials, including all the radical pictures and VAS scores before and after surgery for all the three patients, are included within the article.

## Abbreviations

2D: 2-dimensional
AS: Ankylosing spondylitis
CT: Computed Tomography
MRI: Magnetic Resonance Imaging
PE: Physical examination

## References

[1] Leone A, Marino M, Dell’Atti C, Zecchi V, Magarelli N, Colosimo C (2016). Spinal fractures in patients with ankylosing spondylitis. Rheumatol Int.

[2] Hu X, Thapa AJ, Cai Z, Wang P, Huang L, Tang Y, Ye J, Cheng K, Shen H (2016). Comparison of smith-petersen osteotomy, pedicular subtraction osteotomy, and poly-segmental wedge osteotomy in treating rigid thoracolumbar kyphotic deformity in ankylosing spondylitis a systematic review and meta-analysis. BMC Surg.

[3] Munoz-Ortego J, Vestergaard P, Rubio JB, Wordsworth P, Judge A, Javaid MK, Arden NK, Cooper C, Diez-Perez A, Prieto-Alhambra D (2014). Ankylosing spondylitis is associated with an increased risk of vertebral and nonvertebral clinical fractures: a population-based cohort study. J Bone Miner Res.

[4] Fatemi G, Gensler LS, Learch TJ, Weisman MH (2014). Spine fractures in ankylosing spondylitis: a case report and review of imaging as well as predisposing factors to falls and fractures. Semin Arthritis Rheum.

[5] Miao Jinhao, Chen Yu, Zhang Bangke, Li Tiefeng, Luo Yibing, Shi Lei, Shi Jiangang, Chen Deyu (2018). Surgical Treatment for Odontoid Fractures in Patients with Long-Standing Ankylosing Spondylitis: A Report of 3 Cases and Review of the Literature. World Neurosurgery.

[6] Allouch H., Shousha M., Böhm H. (2017). Operationen bei ankylosierender Spondylitis (Morbus Bechterew). Zeitschrift für Rheumatologie.

[7] El TN, Abode-Iyamah KO, Hitchon PW, Dahdaleh NS (2015). Management of spinal fractures in patients with ankylosing spondylitis. Clin Neurol Neurosurg.

[8] Sapkas G, Kateros K, Papadakis SA, Galanakos S, Brilakis E, Machairas G, Katonis P (2009). Surgical outcome after spinal fractures in patients with ankylosing spondylitis. BMC Musculoskelet Disord.

[9] Wang Y, Zhang Y, Mao K, Zhang X, Wang Z, Zheng G, Li G, Wood KB (2010). Transpedicular bivertebrae wedge osteotomy and discectomy in lumbar spine for severe ankylosing spondylitis. J Spinal Disord Tech.

[10] Kurucan E, Bernstein DN, Mesfin A (2018). Surgical management of spinal fractures in ankylosing spondylitis. J Spine Surg.

[11] Vazan M, Ryang YM, Barz M, Torok E, Gempt J, Meyer B (2019). Ankylosing spinal disease-diagnosis and treatment of spine fractures. World Neurosurg.

[12] Ma J, Wang C, Zhou X, Zhou S, Jia L (2015). Surgical therapy of cervical spine fracture in patients with ankylosing spondylitis. Medicine (Baltimore).

[13] Teunissen FR, Verbeek BM, Cha TD, Schwab JH (2017). Spinal cord injury after traumatic spine fracture in patients with ankylosing spinal disorders. J Neurosurg Spine.

[14] Isogai N, Asamoto S, Nakamura S, Sakurai K, Ishihara S, Ishikawa M, Nishiyama M, Yoshioka F, Samura K, Kawashima M (2018). Spine and spinal cord injury associated with a fracture in elderly patients with ankylosing spondylitis. Neurol Med Chir (Tokyo).

[15] Davey-Ranasinghe N, Deodhar A (2013). Osteoporosis and vertebral fractures in ankylosing spondylitis. Curr Opin Rheumatol.

[16] Pray C, Feroz NI, Nigil HN (2017). Bone mineral density and fracture risk in ankylosing spondylitis: a meta-analysis. Calcif Tissue Int.

[17] Maas F, Spoorenberg A, van der Slik B, van der Veer E, Brouwer E, Bootsma H, Bos R, Wink FR, Arends S (2017). Clinical risk factors for the presence and development of vertebral fractures in patients with ankylosing spondylitis. Arthritis Care Res (Hoboken).

[18] Zhu R, Song W, Hu W, Jiang Z, Yuan J, Cui Z, Wan J, Liu Y, Feng S, Zhang X (2017). The treatment strategies for spine fractures in patients with ankylosing spondylitis: a case report. Medicine (Baltimore).

[19] Jacobs WB, Fehlings MG (2008). Ankylosing spondylitis and spinal cord injury: origin, incidence, management, and avoidance. Neurosurg Focus.

[20] Qiao M, Qian BP, Qiu Y, Mao SH, Wang YH (2019). Radiologic and pathological investigation of Pseudarthrosis in ankylosing spondylitis: distinguishing between inflammatory and traumatic etiology. J Rheumatol.

[21] Werner BC, Samartzis D, Shen FH (2016). Spinal fractures in patients with ankylosing spondylitis: etiology, diagnosis, and management. J Am Acad Orthop Surg.

[22] Stenhouse G, Ulbricht C, Khanna M (2014). Spinal injury in ankylosing spondylitis. BMJ.

[23] Robinson Y, Willander J, Olerud C (2015). Surgical stabilization improves survival of spinal fractures related to ankylosing spondylitis. Spine (Phila Pa 1976).

[24] Westerveld LA, Verlaan JJ, Oner FC (2009). Spinal fractures in patients with ankylosing spinal disorders: a systematic review of the literature on treatment, neurological status and complications. Eur Spine J.

[25] Liu R, Sun L, Li CH, Zhai JY, Liu XY (2017). Cause analysis of spinal surgery in ankylosing spondylitis. Beijing Da Xue Xue Bao.

[26] Rustagi T, Drazin D, Oner C, York J, Schroeder GD, Vaccaro AR, Oskouian RJ, Chapman JR (2017). Fractures in spinal ankylosing disorders: a narrative review of disease and injury types, treatment techniques, and outcomes. J Orthop Trauma.

[27] Whang PG, Goldberg G, Lawrence JP, Hong J, Harrop JS, Anderson DG, Albert TJ, Vaccaro AR (2009). The management of spinal injuries in patients with ankylosing spondylitis or diffuse idiopathic skeletal hyperostosis: a comparison of treatment methods and clinical outcomes. J Spinal Disord Tech.

[28] Bredin S, Fabre-Aubrespy M, Blondel B, Falguieres J, Schuller S, Walter A, Fuentes S, Tropiano P, Steib JP, Charles YP (2017). Percutaneous surgery for thoraco-lumbar fractures in ankylosing spondylitis: study of 31 patients. Orthop Traumatol Surg Res.

[29] Kruger A, Frink M, Oberkircher L, El-Zayat BF, Ruchholtz S, Lechler P (2014). Percutaneous dorsal instrumentation for thoracolumbar extension-distraction fractures in patients with ankylosing spinal disorders: a case series. Spine J.

[30] Yeoh D, Moffatt T, Karmani S (2014). Good outcomes of percutaneous fixation of spinal fractures in ankylosing spinal disorders. Injury.

